# Structural mechanism for guanylate-binding proteins (GBPs) targeting by the *Shigella* E3 ligase IpaH9.8

**DOI:** 10.1371/journal.ppat.1007876

**Published:** 2019-06-19

**Authors:** Chenggong Ji, Shuo Du, Peng Li, Qinyu Zhu, Xiaoke Yang, Chunhong Long, Jin Yu, Feng Shao, Junyu Xiao

**Affiliations:** 1 The State Key Laboratory of Protein and Plant Gene Research, School of Life Sciences, Peking-Tsinghua Center for Life Sciences, Peking University, Beijing, China; 2 National Institute of Biological Science (NIBS), Beijing, China; 3 Beijing Computational Science Research Center, Beijing, China; University of California San Diego, UNITED STATES

## Abstract

The guanylate-binding proteins (GBPs) belong to the dynamin superfamily of GTPases and function in cell-autonomous defense against intracellular pathogens. IpaH9.8, an E3 ligase from the pathogenic bacterium *Shigella flexneri*, ubiquitinates a subset of GBPs and leads to their proteasomal degradation. Here we report the structure of a C-terminally truncated GBP1 in complex with the IpaH9.8 Leucine-rich repeat (LRR) domain. IpaH9.8^LRR^ engages the GTPase domain of GBP1, and differences in the Switch II and α3 helix regions render some GBPs such as GBP3 and GBP7 resistant to IpaH9.8. Comparisons with other IpaH structures uncover interaction hot spots in their LRR domains. The C-terminal region of GBP1 undergoes a large rotation compared to previously determined structures. We further show that the C-terminal farnesylation modification also plays a role in regulating GBP1 conformation. Our results suggest a general mechanism by which the IpaH proteins target their cellular substrates and shed light on the structural dynamics of the GBPs.

## Author summary

*Shigella flexneri* is a Gram-negative bacteria that causes diarrhea in humans and leads to a million deaths every year. Once inside the cell, *S*. *flexneri* injects the host cell cytoplasm with effector proteins to suppress host defense. The guanylate-binding proteins (GBPs) have potent antimicrobial functions against a number of pathogens including *S*. *flexneri*. For successful infection, *S*. *flexneri* relies on an effector protein known as IpaH9.8, a unique ubiquitin E3 ligase to target a subset of GBPs for proteasomal degradation. How these GBPs are specifically recognized by IpaH9.8 was unclear. Here, using a combination of structural and biochemical approaches, we reveal the molecular basis of GBP-IpaH9.8 interaction, and show that subtle differences in the seven human GBPs can significantly impact the targeting specificity of IpaH9.8. We also show that the GBPs have considerable structural flexibility, which is likely important for their function. Our results provide further insights into *S*. *flexneri* pathogenesis, and laid the groundwork for future biophysical and biochemical studies to investigate the functional mechanism of GBPs.

## Introduction

The guanylate-binding proteins (GBPs) play critical roles in cell-autonomous immunity against a diverse range of bacterial, viral, and protozoan pathogens. The charter member of this family is GBP1, which was identified as a protein that is strongly induced by the interferons and can specifically bind to the guanylate affinity column [1, 2]. There are seven GBPs in human (GBP1-7), which share 52%-88% sequence identity between each other [3]. GBP1, GBP2, and GBP5 contain C-terminal CaaX box sequences that allow them to be prenylated in cells. GBP1 is farnesylated, which is important for its localization to membrane structures such as the Golgi apparatus [4, 5]. The farnesylation modification, together with a nearby triple-arginine motif, is also required for the localization of GBP1 to cytosolic bacteria [6, 7]. Once on the bacterial surface, GBP1 is able to recruit other GBPs via heterodimerization and oligomerization [7, 8]. A unique property of GBP1 is its ability to hydrolyze GTP first to GDP and then to GMP in a processive manner [9, 10]. In contrast, GBP2 only converts ~10% GTP to GMP, whereas GBP5 hydrolyzes GTP only to GDP [11, 12]. The physiological significance of the unusual enzyme activity of GBP1, as well as the biochemical differences between different GBPs, remains unclear. Mechanistically, the GBPs belong to the dynamin superfamily of GTPases, which often mediate membrane fission or fusion [13, 14]. By analogy, the GBPs could also function in the membrane remodeling processes. For example, they may contribute to the lysis of pathogen-containing vacuoles. Other reported functions of the GBPs include promoting autophagy, initiating inflammasome assembly, and inhibiting bacterial motility (for recent reviews, see [15–20]). However, our understanding towards the functions of these important proteins is still in its infancy.

The GBPs have complex structural dynamics. Crystal structures have been determined for the full-length GBP1 in the monomer state and the isolated GTPase domain of GBP1 in the dimer state [10, 21, 22]. GBP1 contains an N-terminal large GTPase (LG) domain and a C-terminal helical region, which can be further divided into a middle domain (MD) that contains the α7-α11 helices and a GTPase effector domain (GED) that consists of the α12-α13 helices. The GED folds back and interacts with LG and MD, which is important to maintain GBP1 at the resting state [21, 23]. Binding of GTP induces the release of GED from the rest of the protein, resulting in an extended conformation that was previously interpreted as a “dimer” based on size-exclusion chromatography analyses [24]. Unlike the isolated LG domain that readily dimerizes under several guanine nucleotide conditions, full-length GBP1 only forms a stable dimer in the presence of GDP-AlFx that mimics the catalytic transition state [10, 24]. Due to the extended conformation of the GED domain, the dimer of the full-length protein has a large hydrodynamic radius and was long regarded as a “tetramer”. Dimerized full-length GBP1 can cause the tethering of unilamellar vesicles *in vitro*, and this activity depends on the C-terminal farnesylation modification [25]. Furthermore, the farnesylated GBP1 can form a transient ring-like oligomer that is reminiscent of dynamin and related proteins such as the Mx (Myxovirus resistance) proteins [25]. Whether these properties are related to the cellular functions of GBP1 remains to be investigated.

Pathogens often antagonize key cellular proteins to evade host defense. Due to the important functions of the GBPs in innate immunity, it is not a surprise that some pathogens have evolved strategies to counter their activity. The IpaH family of proteins are unique E3 ubiquitin ligases that are only found in bacteria, especially pathogenic bacteria such as *Shigella* and *Salmonella* [26]. They all contain an N-terminal Leucine-rich repeat (LRR) domain and a C-terminal novel E3 ligase (NEL) domain. Although the NEL domain is structurally unrelated to the HECT family of E3 ligases, it also catalyzes the ubiquitination reaction by forming a ubiquitin-thioester intermediate via an invariant Cys in the CxD motif [27, 28]. IpaH9.8 from *Shigella flexneri*, an intracellular bacterium that causes bacillary dysentery, is one of the most extensively studied member of the IpaH family. In fact, it is one of the first IpaH proteins that is demonstrated to be an E3 ligase [26]. Recent studies have discovered that IpaH9.8 ubiquitinates and degrades a subset of GBPs, which is important for *S*. *flexneri* to suppress host defense and replicate in the cells [6–8].

To delineate how the GBPs are targeted by IpaH9.8 and gain further insights into GBP-mediated immunity, we have first determined the crystal structure of IpaH9.8^LRR^ in complex with GBP1^LG-MD^, which explains the specific recognition of select GBPs by IpaH9.8. Mutating the GBP1-binding residues in IpaH9.8 diminish its ability to degrade the GBPs. By comparing with other IpaH protein structures, we have identified interaction hot spots in the LRR domains of this unique family of bacterial ubiquitin ligases. A large rotation of GBP1^MD^ is observed in our structure, revealing that the elastic α7 helix plays an important role in regulating the structural dynamics of GBP1. Finally, we determined the structure of farnesylated full-length GBP1 and show that the farnesylation modification is involved in restraining GBP1 conformation.

## Results

### Overall structure of the GBP1^LG-MD^/IpaH9.8^LRR^ complex

**Fig 1:**
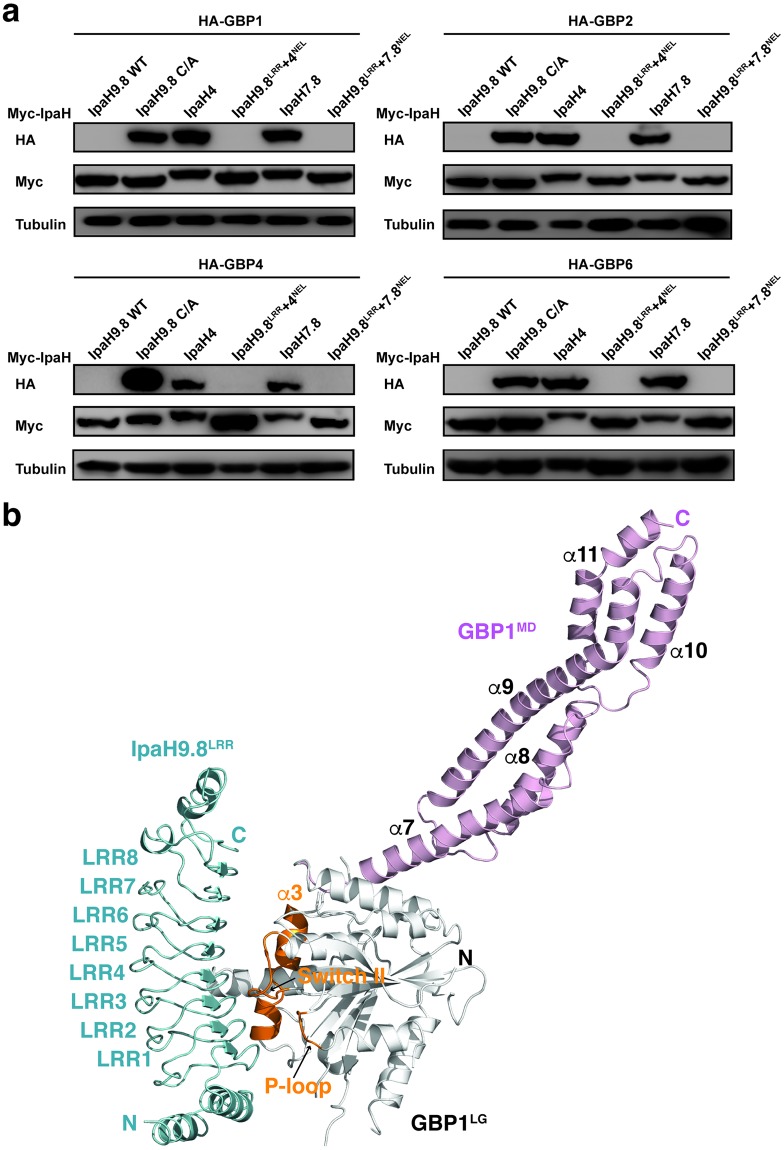
The LRR domain of IpaH9.8 dictates substrate specificity. (**a**) HA tagged GBPs were co-expressed with 6xMyc tagged IpaH proteins in HEK293T cells, and then the cell lysates were immunoblotted with HA and Myc antibodies. C/A indicates IpaH9.8-C337A, an enzyme dead mutant of IpaH9.8. IpaH9.8^LRR^+4^NEL^ and IpaH9.8^LRR^+7.8^NEL^ are two chimera IpaH proteins that contain the N-terminal LRR domain of IpaH9.8 (residues 1–247) and the C-terminal NEL domains of IpaH4 (residues 276–583) and IpaH7.8 (residues 258–565), respectively. (**b**) Overall structure of the GBP1^LG-MD^/IpaH9.8^LRR^ complex. GBP1^LG^ (residues 1–308) is mainly shown in white, with the P-loop, Switch II, and α3 helix regions highlighted in orange. GBP1^MD^ (residues 308–479) is shown in light magenta. IpaH9.8^LRR^ is shown in cyan. The N and C-termini of both molecules are indicated.

The IpaH proteins are modular enzymes that all contain a LRR domain and a NEL domain. The NEL domains are highly conserved, and therefore the substrate specificity is largely dictated by the variable LRR domains. Indeed, IpaH9.8^LRR^ binds to GBP1 [6]. Swapping the LRR domains of IpaH4 and IpaH7.8 to IpaH9.8^LRR^ enables the chimera IpaHs to degrade the GBPs (Fig 1a).

To elucidate the molecular basis of how IpaH9.8^LRR^ recognizes GBP1, we sought to determine their complex structure. We first crystallized full-length GBP1 in complex with IpaH9.8^LRR^. However, the crystal diffracted to only ~10 Å and could not be improved despite extensive attempts. We subsequently crystallized the LG-MD region of GBP1 (GBP1^LG-MD^) in complex with IpaH9.8^LRR^ and determined the structure at 3.6 Å (Table 1, Fig 1b). The moderate resolution is likely caused by a high solvent content of the crystal (73.4%). Nevertheless, the electron density map generated from the molecular replacement solution is of high quality and allows unambiguous model building (S1 Fig).

**Table 1:** Data collection and refinement statistics.

	GBP1^LG-MD^/IpaH9.8^LRR^	GBP1_F_
**Data collection**		
Space group	P 3_1_ 2 1	P 2_1_ 2_1_ 2_1_
Cell dimensions	a = 211.7 Å, b = 211.7 Å, c = 57.0 Å	a = 48.2 Å, b = 114.0 Å, c = 127.5 Å
Resolution (Å)	3.60	2.30
*I* / σ*I*	12.0 (1.8)	28.5 (2.3)
*R*_merge_	0.196 (2.895)	0.090 (1.043)
*R*_pim_	0.068 (0.992)	0.027 (0.349)
CC1/2	0.945 (0.706)	0.999 (0.978)
Completeness (%)	100.0 (100.0)	99.5 (99.7)
Multiplicity	9.8 (9.4)	11.9 (8.8)
**Refinement**		
Reflections used in refinement	14485 (451)	24813 (1091)
Reflections used for *R*_free_	730 (23)	2000 (88)
*R*_work_ / *R*_free_	0.240 / 0.287	0.220 / 0.267
No. of non-hydrogen atoms		
Protein	5138	4388
Ligands	0	15
Solvent	0	128
Protein residues	642	545
*B*-factors		
Protein	60.7	39.89
Ligands		40.46
Solvent		35.69
R.m.s deviations		
Bond lengths (Å)	0.003	0.005
Bond angles (°)	0.57	0.97
Ramachandran		
Favored (%)	86.15	97.37
Allowed (%)	12.90	2.07
Outliers (%)	0.96	0.56

Each dataset was collected from a single crystal. Values in parentheses are for highest-resolution shell.

The LG domain of GBP1 features a canonical globular GTPase fold that highly resembles GBP1^LG^ in the full-length GBP1 structure [21, 22]. Superimposing it to the full-length structure generates a root mean square deviation (rmsd) of 1.0 Å for 257 Cα atoms. The MD domain features two three-helix bundles that spiral around the common α9 helix and also resembles the corresponding region in the full-length structure. Superimposing the MD domain from our structure to the corresponding region in full-length GBP1 yields a rmsd of 1.9 Å for 169 Cα atoms. However, the arrangement of the LG and MD in our structure is different from that in the full-length structure, and a large swing of the MD is observed (S2a Fig). IpaH9.8^LRR^ is very similar to the previously determined IpaH9.8^LRR^ alone structure [29], and contains eight LRR motifs (LRR1-LRR8) that are organized into a slightly curved solenoid. In the complex structure, it engages GBP1^LG^ using the concave surface of the solenoid (Fig 1b). Three regions in GBP1^LG^ are involved in interacting with IpaH9.8^LRR^: the P-loop, the switch II region, and the α3 helix (Fig 1b). These regions are located on the opposite side of the GED domain in the full-length GBP1 structure, so the GED domain, which is not present in our structure, would not interfere with the binding (S2a Fig). On the other hand, these regions are involved in forming the dimer interface in the LG dimer structure [10], and therefore binding of IpaH9.8 would lead to the disruption of the GBP1^LG^ dimer (S2b Fig). This is consistent with our previous observation that IpaH9.8 disrupts the GBP1 “tetramer” in the presence of GDP-AlFx [6].

### The GBP1^LG-MD^/IpaH9.8^LRR^ interface

**Fig 2:**
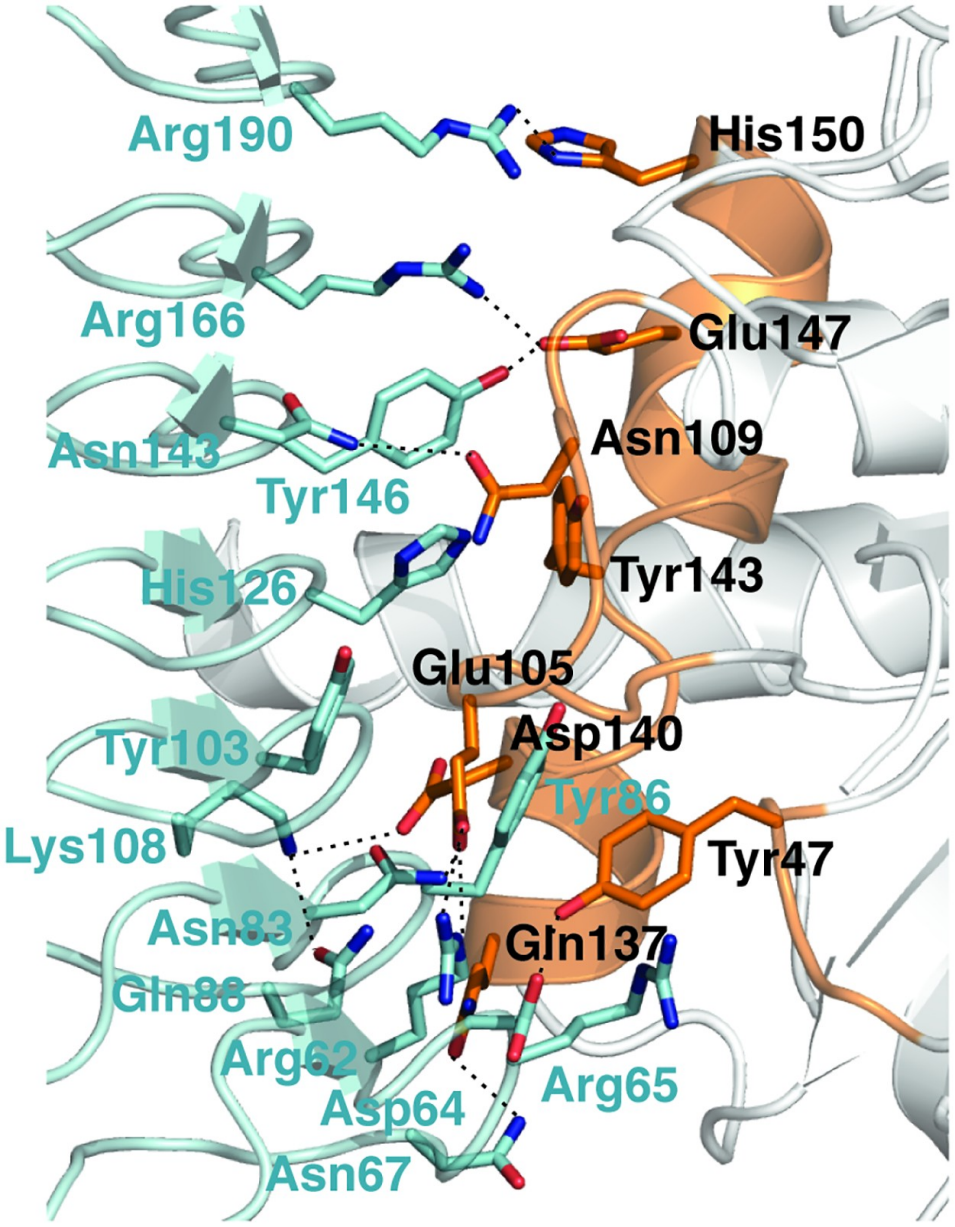
The GBP1^LG-MD^/IpaH9.8^LRR^ interface. Detailed view of the GBP1^LG-MD^/IpaH9.8^LRR^ interface. GBP1 and IpaH9.8 are shown using the same color scheme as in Fig 1. Hydrogen bond and salt bridge interactions are indicated by dashed lines.

In the structure, seven out of the eight LRR modules in IpaH9.8^LRR^ contribute residues to interact with GBP1 (S1b Fig, Fig 2). In LRR1, Arg62^9.8^ (superscripts 9.8 and G indicate residues in IpaH9.8 and GBP1, respectively) forms bidentate interactions with Glu105^G^ in the Switch II region of GBP1. Asp64^9.8^ forms a hydrogen bond with Tyr47^G^, and Arg65^9.8^ interacts with Tyr47^G^ via a cation-π interaction. Asn67^9.8^ forms a hydrogen bond with Gln137^G^. In LRR2, Asn83^9.8^ forms a hydrogen bond with Glu105^G^.Tyr86^9.8^ forms a hydrogen bond with the main chain carbonyl group of Gly102^G^, at the same time forms van der waals interactions with Tyr47^G^. Gln88^9.8^ appears to stabilize the position of Lys108^9.8^ in LRR3, which in turn forms a salt bridge with Asp140^G^. Other residues in LRR3 that interact with GBP1 include Tyr103^9.8^, which packs against the aliphatic region of Glu105^G^. His126^9.8^ from LRR4 interacts with Tyr143^G^ via cation-π and van der waals interactions. In LRR5, Asn143^9.8^ forms a hydrogen bond with Asn109^G^, and Tyr146^9.8^ hydrogen bonds with Glu147^G^. Arg166^9.8^ from LRR6 forms a salt bridge with Glu147^G^. Arg190^9.8^ from LRR7 may form a hydrogen bond with His150^G^. The residues involved in binding GBP1 are unique to IpaH9.8 (S3 Fig), explaining the fact that only IpaH9.8, but not other IpaH proteins, specifically degrades the GBPs [6, 8].

**Fig 3:**
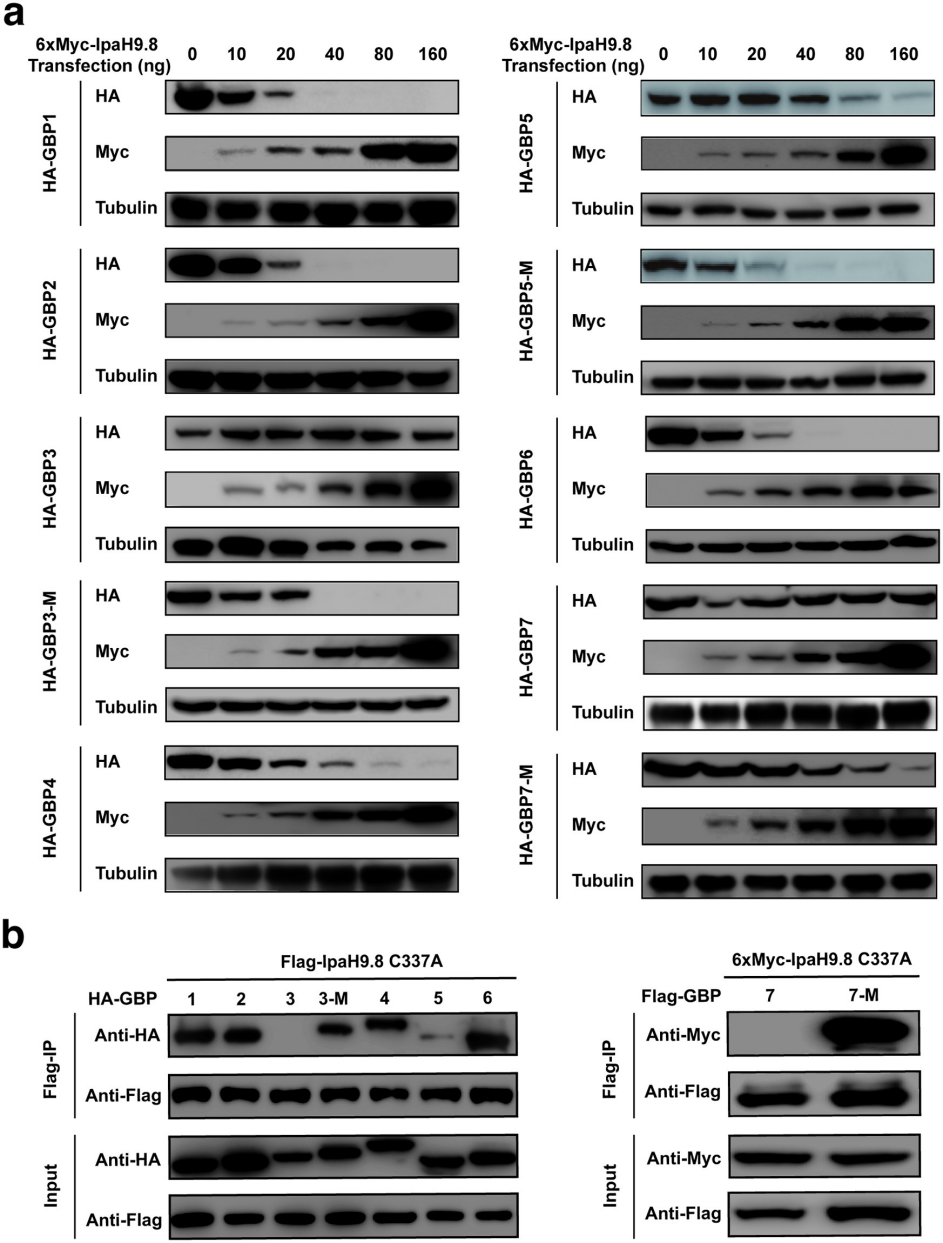
GBP3, GBP5, and GBP7 mutants are more efficiently degraded by IpaH9.8. (**a**) 1 μg HA or Flag tagged GBPs were co-expressed with increasing amounts of 6xMyc tagged IpaH9.8 in HEK293T cells as indicated. The cell lysates were analyzed by immunoblotting with indicated antibodies. The GBP3, GBP5, and GBP7 mutants are more susceptible to IpaH9.8 mediated degradation compared to the wild-type proteins. The data are representatives of three independent experiments. (**b**) GBP3 and GBP7 mutants display enhanced interactions with IpaH9.8-C337A. HA or Flag tagged GBPs were co-expressed with Flag or Myc tagged IpaH-C337A in HEK293T cells. Immunoprecipitation was performed using the Flag-M2 beads from the cell lysates, and associated proteins were analyzed by immunoblotting.

The seven human GBPs are highly homologous to each other. However, only a subset of GBPs such as GBP1, GBP2, GBP4, and GBP6 are efficiently targeted and degraded by IpaH9.8 [6, 8]. GBP3 and GBP7 are particularly resistant (Fig 3a). Careful examination reveals subtle differences in their Switch II and α3 helix regions. For example, GBP3 contains a Lys (Lys105) in its Switch II that aligns with Glu105 in GBP1 (S4 Fig), which lies at the center of GBP1^LG-MD^/IpaH9.8^LRR^ interface and makes critical interactions with several IpaH9.8 residues (Fig 2). Mutation of this residue to Glu allows the GBP3 mutant (GBP3-M) to be efficiently degraded by IpaH9.8 (Fig 3a). GBP3-M also binds strongly to IpaH9.8-C337A, an IpaH9.8 mutant that has abolished E3 ligase activity (Fig 3b). The α3 helix of GBP5 is slightly different when compared with GBP1 (S4 Fig). Gly137, Leu141, and His143 replace GBP1 residues Gln137, Gln141, and Tyr143, respectively. These differences likely reduce the interaction between GBP5 and IpaH9.8, and make GBP5 a suboptimal substrate that requires higher amounts of IpaH9.8 for degradation (Fig 3a). A double mutant of GBP5, G137Q/L141Q (GBP5-M), is degraded more efficiently by IpaH9.8 (Fig 3a). Several residues in the Switch II and α3 helix region of GBP7 are different compared to GBP1, including Met104 that replaces Val104 in GBP1 and His143 like in GBP5 (S4 Fig). The bulkier Met104 may hinder the binding of IpaH9.8. Furthermore, molecular dynamics simulation study suggests that the α3 helix region of GBP7 prefers to adopt a loop rather than a helical conformation (S5 Fig), caused partly by the presence of Ser111, instead of an Asn in other GBPs, at the end of its Switch II (S4 Fig). Ser111 appears to stabilize a hydrogen bond interaction between Ser113 and Glu147, which causes the α3 helix to unfold. Swapping the GBP7 Switch II-α3 region (residues 104–151) to the corresponding segment in GBP1 renders the GBP7 mutant (GBP7-M) susceptible to IpaH9.8-mediated degradation (Fig 3a). GBP7-M also shows a stronger interaction with IpaH9.8-C337A (Fig 3b).

### Mutations in IpaH9.8^LRR^ diminish IpaH9.8 function

**Fig 4:**
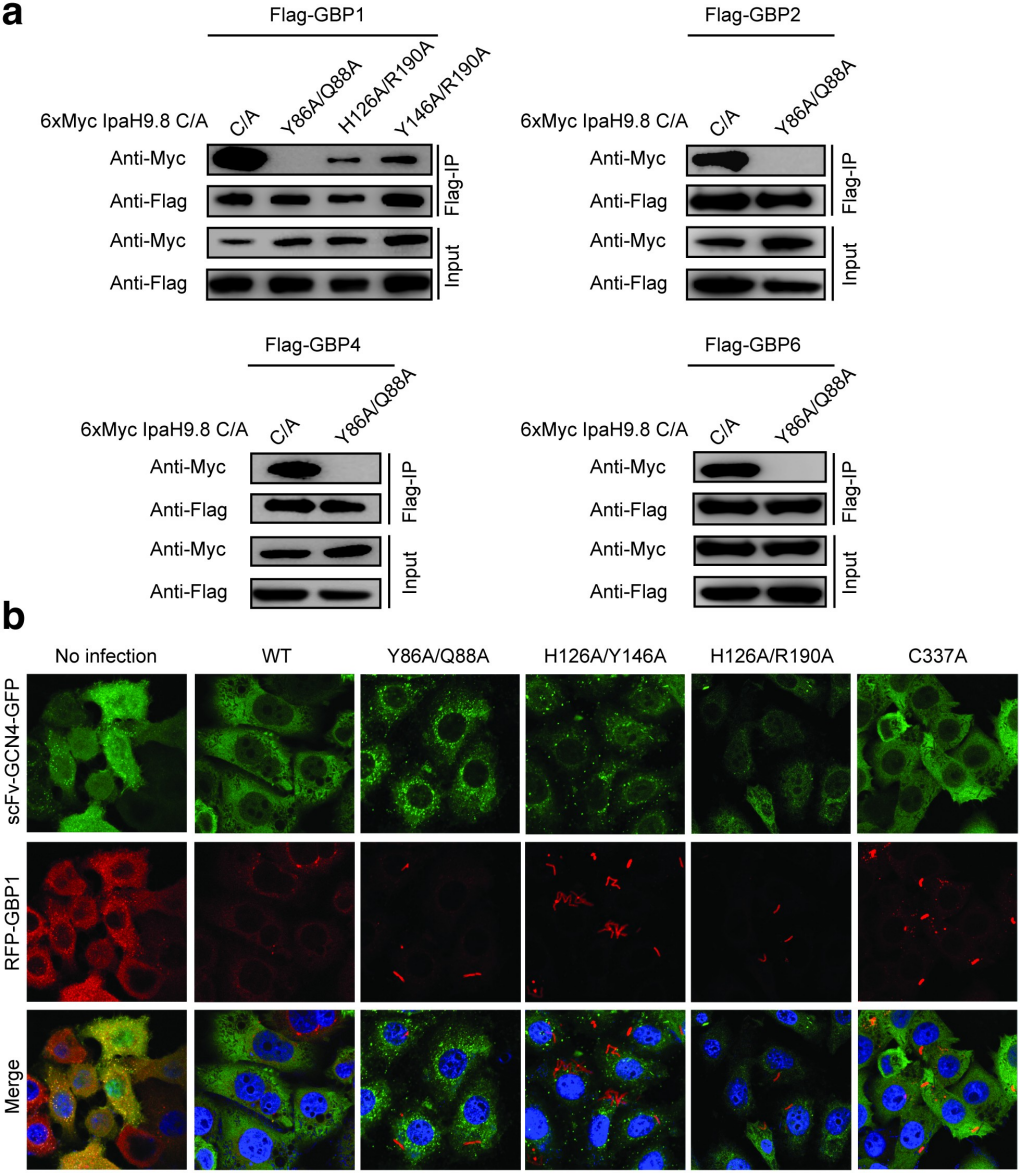
IpaH9.8 mutants display decreased activity to degrade the GBPs. (**a**) IpaH9.8 mutants show reduced interaction with the GBPs. Flag tagged GBPs were co-expressed with 6xMyc tagged IpaH-C337A in HEK293T cells. The GBPs were immunoprecipitated from the cell lysates using the Flag-M2 beads, and associated IpaH was detected by immunoblotting. (**b**) Degradation of RFP-GBP1 by different IpaH9.8 mutants. HeLa cells stably expressing RFP-GBP1 and scFV-Suntag-GFP were infected with *S*. *flexneri ΔipaH9*.*8* expressing various IpaH9.8 proteins fused to 10xSunTags. In uninfected cells, GCN4-GFP (green) displays a dispersed pattern in the cell. Infection of *S*. *flexneri* expressing wild-type IpaH9.8-10xSunTag leads to the enrichment of GFP signal in the cytoplasm, due to the delivery of IpaH9.8 by the bacteria and recognition of the SunTags by GCN4. The RFP signal (red) is largely diminished due to the degradation of GBP1 by IpaH9.8. In contrast, IpaH mutants have impaired functions to degraded GBP1, resulting in the recruitment of RFP-GBP1 onto the bacteria. Nuclei were stained with DAPI (blue). The data are representatives of two independent experiments.

To further verify our structure, we mutated several IpaH residues that are involved in binding to GBP1, including Tyr86, Gln88, His126, Tyr146, and Arg190. When these mutations are generated in combination with C337A, the resulting mutants IpaH9.8-Y86A/Q88A/C337A, IpaH9.8-H126A/R190A/C337A, and IpaH9.8-Y146A/R190A/C337A all display greatly reduced interaction with GBP1, as shown by the co-immunoprecipitation experiments (Fig 4a). Mutating Tyr86 and Gln88 together generates the strongest effect. Similarly, IpaH9.8-Y86A/Q88A/C337A also failed to interact with other GBPs, including GBP2, GBP4, and GBP6 (Fig 4a).

To validate the physiological relevance of these GBP-binding residues, we performed cell imaging experiments as we previously described [6]. We made mutations to IpaH9.8 that are fused with 10 tandem repeats of the SUperNova tags (SunTags) [30]. We then expressed these IpaH9.8 mutants in the *S*. *flexneri ΔipaH9*.*8* strain and used these bacteria to infect HeLa cells stably expressing RFP-GBP1 and scFv-GCN4-GFP. GCN4 is a single chain antibody that specifically recognizes the SunTag. In uninfected cells, GCN4-GFP display a dispersed pattern in the cell (Fig 4b). When infected with *S*. *flexneri* expressing wild-type IpaH9.8-10xSunTag, the GFP signals are enriched in the cytoplasm due to the delivery of IpaH9.8 by the bacteria, and the RFP signal is largely diminished due to the degradation of GBP1 (Fig 4b). In contrast, RFP-GBP1 is not efficiently degraded by the bacterial strains expressing IpaH9.8-Y86A/Q88A, IpaH9.8-H126A/Y146A, or IpaH9.8-Y146A/R190A. In these cells, the RFP signal is most bright around the bacteria, due to the localization of GBP1 to the bacterial surface (Fig 4b). Together, these results demonstrate that an intact GBP-binding surface in IpaH9.8^LRR^ is critical for the function of IpaH9.8 *in vivo*.

### Interaction hot spots in the LRR domains of IpaH proteins

**Fig 5:**
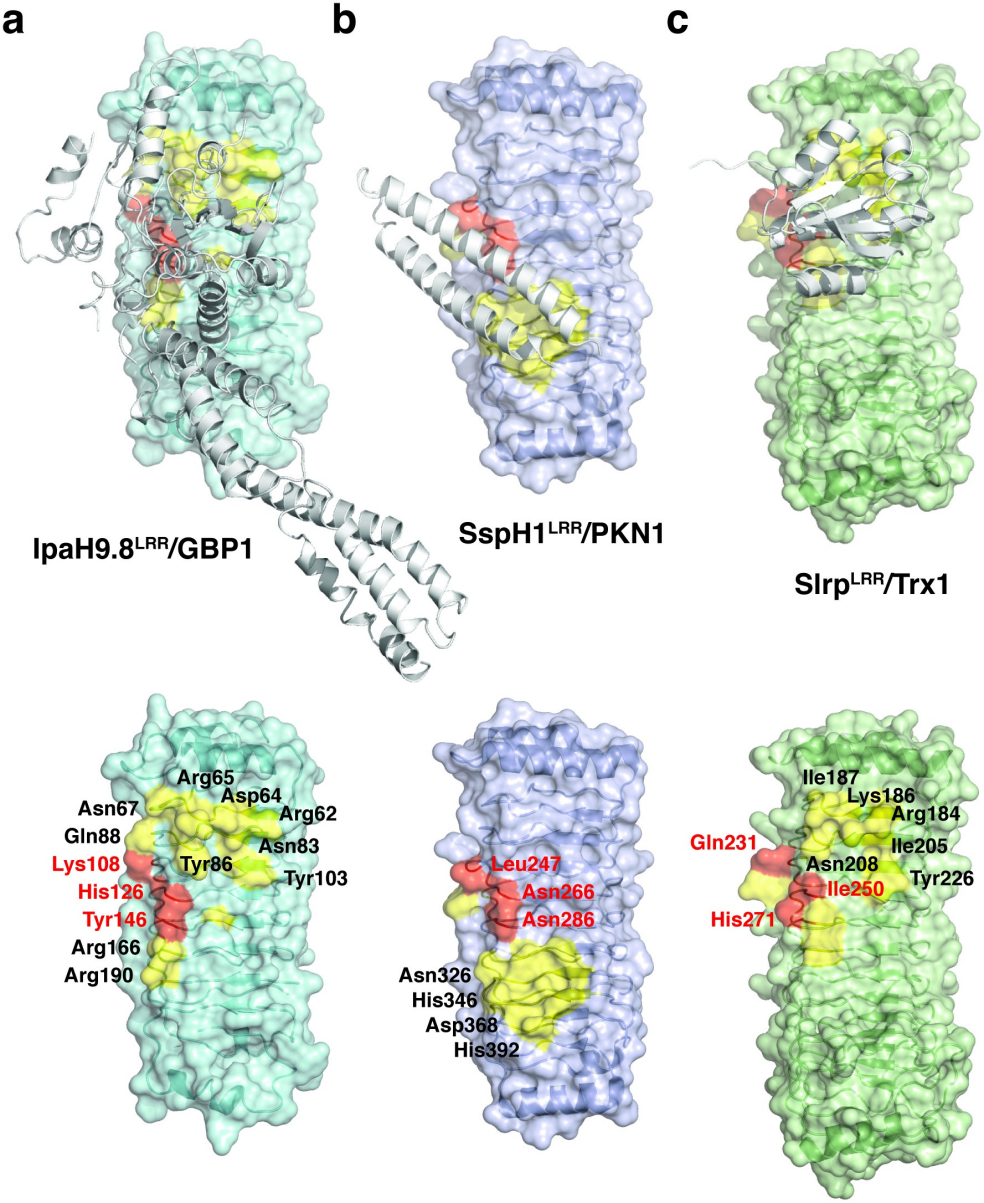
Interaction hot spots in the LRR domains of the IpaH family proteins. (**a**) The structure of IpaH9.8^LRR^ in complex with GBP1. IpaH9.8^LRR^ is shown as colored ribbon diagrams enclosed in its van der Waals surface. GBP1 is shown as white ribbons. IpaH9.8 residues involved in binding to GBP1 are colored in yellow, and the three hot spot residues are highlighted in red. A IpaH9.8^LRR^ alone structure without GBP1 is shown at the bottom for the GBP1-binding residues to be clearly seen. **(b**) The structure of SspH1^LRR^ in complex with a coiled-coil region of the PKN1 kinase (PDB ID: 4NKG). SspH1^LRR^ is shown as colored ribbon diagrams in its van der Waals surface, and PKN1 is shown as white ribbons. **(c**) Type I binding site in the structure of Slrp^LRR^ in complex with human Trx1 thioredoxin (PDB ID: 4PUF). Slrp^LRR^ is shown as colored ribbon diagrams in its van der Waals surface, and Trx1 is shown as white ribbons.

The IpaH proteins have diverse substrates in the host [31]. In particular, two IpaH proteins from *Salmonella*, SspH1 and Slrp, use their LRR domains to target the host PKN1 kinase and Trx1 thioredoxin, respectively [32, 33]. Crystal structures have been determined for SspH1^LRR^ in complex with a coiled-coil region of the PKN1 kinase [34], and Slrp in complex with Trx1 [35]. Comparing these structures with the GBP1^LG-MD^/IpaH9.8^LRR^ complex reveals both differences and common features (Fig 5).

Like IpaH9.8, SspH1 binds its target using the concave surface of its LRR domain. While the N-terminal region of IpaH9.8^LRR^ mediates the majority of the interactions with GBP1, the contact site for PKN1 is more focused on the C-terminal half of SspH1^LRR^ (Fig 5a and 5b). Nonetheless, the edge of the concave surface that are pointed by the LRR strands are important for the binding in both structures. In IpaH9.8^LRR^, Asn67 from LRR1, Gln88 from LRR2, Lys108 from LRR3, His126 from LRR4, Tyr146 from LRR5, Arg166 from LRR6, and Arg190 from LRR7 form a continuous surface patch that are critical for GBP1 binding (Fig 5a). In SspH1^LRR^, a similar edge is formed by Leu247 from LRR3, Asn266 from LRR4, Asn286 from LRR5, Asn326 from LRR7, His346 from LRR8, Asp368 from LRR9, and His392 from LRR10 (Fig 5b). When SspH1^LRR^ is compared with IpaH9.8^LRR^, SspH1 residues Leu247, Asn266, Asn286, and Asn326 align exactly with IpaH9.8 residues Lys108, His126, Tyr146, and Arg190, respectively (S3 Fig).

In the crystal structure of Slrp/Trx1, Slrp interacts with Trx1 using two interfaces [35]. The so-called type I binding site highly resembles the GBP1 binding site in IpaH9.8^LRR^ (Fig 5a and 5c). This site is formed by the first six LRR modules of Slrp^LRR^, and also involves the concave surface. Trx1 binding residues Arg184, Lys186, Ile187, Ile205, Asn208, Tyr226, Gln231, Ile250, and His271 all align with IpaH9.8 residues Arg62, Asp64, Arg65, Asn83, Tyr86, Tyr103, Lys108, His126, and Tyr146 (S3 Fig). Although the physiological significance of the type I binding site in Slrp remains to be explored, these analyses suggest that the IpaH family proteins could generally bind their target proteins using the LRR concave surfaces. In particular, residues located at positions corresponding to Lys108 in IpaH9.8-LRR3, His126 in IpaH9.8-LRR4, and Tyr146 in IpaH9.8-LRR5 are important for binding in all three complexes (Fig 5, S3 Fig), suggesting that these three positions could function as “hot spots” to mediate the interaction between the IpaH proteins and their cellular targets.

### Conformational change of GBP1

**Fig 6:**
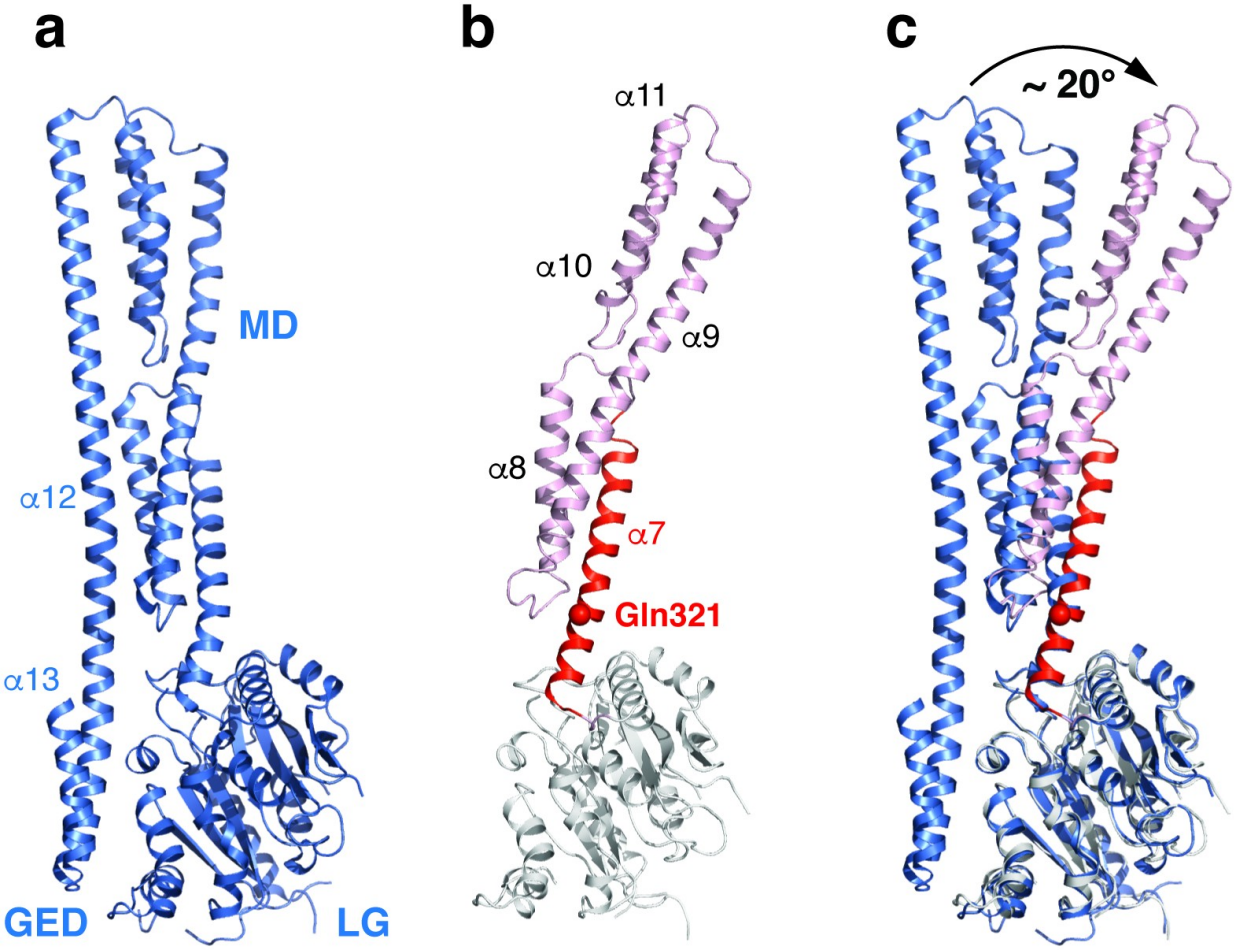
Conformational change of GBP1. (**a**) The structure of full-length GBP1 (PDB ID: 1DG3) is shown as blue ribbons. (**b**) The structure of GBP1^LG-MD^ in the GBP1^LG-MD^/IpaH9.8^LRR^ complex is shown using the same scheme as in Fig 1, except for the α7 helix that is highlighted in red. The Cα atom of Gln321 is shown as a red sphere. (**c**) The LG domain of GBP1^LG-MD^ is superimposed onto the corresponding region in full-length GBP1. The rotation of the MD domain relative to LG is indicated by a curved arrow.

The dynamin superfamily proteins are considered mechanochemical enzymes that convert the energy from GTP binding and hydrolysis to mechanical force. The conformational dynamics of GBP1 is likely at the heart of its function but remains poorly understood. In the previously determined structures, the GED folds back and locks the conformation of GBP1 (Fig 6a). However, biophysical studies suggest that the GED domain is unleashed during the GTPase reaction cycle and the C-terminal region of GBP1 undergoes large degree of conformational change. In our structure, since the GED domain is not present, the MD domain is free to adopt a relaxed conformation. The α7 helix, which is forced to bend in the apo structure due to the interaction between the GED and the LG-MD domains, springs back to the straightened state (Fig 6b). Starting from a highly conserved Gln321 (S4 Fig), the C-terminal half of the α7 helix rotates ~13°, and this conformational change is transmitted toward the rest of the protein, causing an ~20° *en bloc* rotation of the α8-α11 helices (Fig 6c, S2a Fig). Due to the unfavorable geometry of the α7 helix in the “GED on” state, this conformational change likely also occurs in the full-length protein when the GED domain is set free during GBP1 function.

### Structure of farnesylated GBP1

The conformational change seen above prompted us to further investigate the conformation dynamics of GBP1. GBP1 is farnesylated at Cys589, and this modification is important for its localization to the Golgi apparatus and recruitment by various pathogens [4–7]. Despite this modification, GBP1 is primarily a cytosolic protein until the cells are infected by pathogens [4, 5], suggesting that the farnesyl group is probably not exposed at the resting state. The farnesylation modification changes the behavior of GBP1 on hydrophobic chromatography column and reduced its ability to hydrolyze GTP to GMP, suggesting that it impacts the conformation of GBP1 [36].

**Fig 7:**
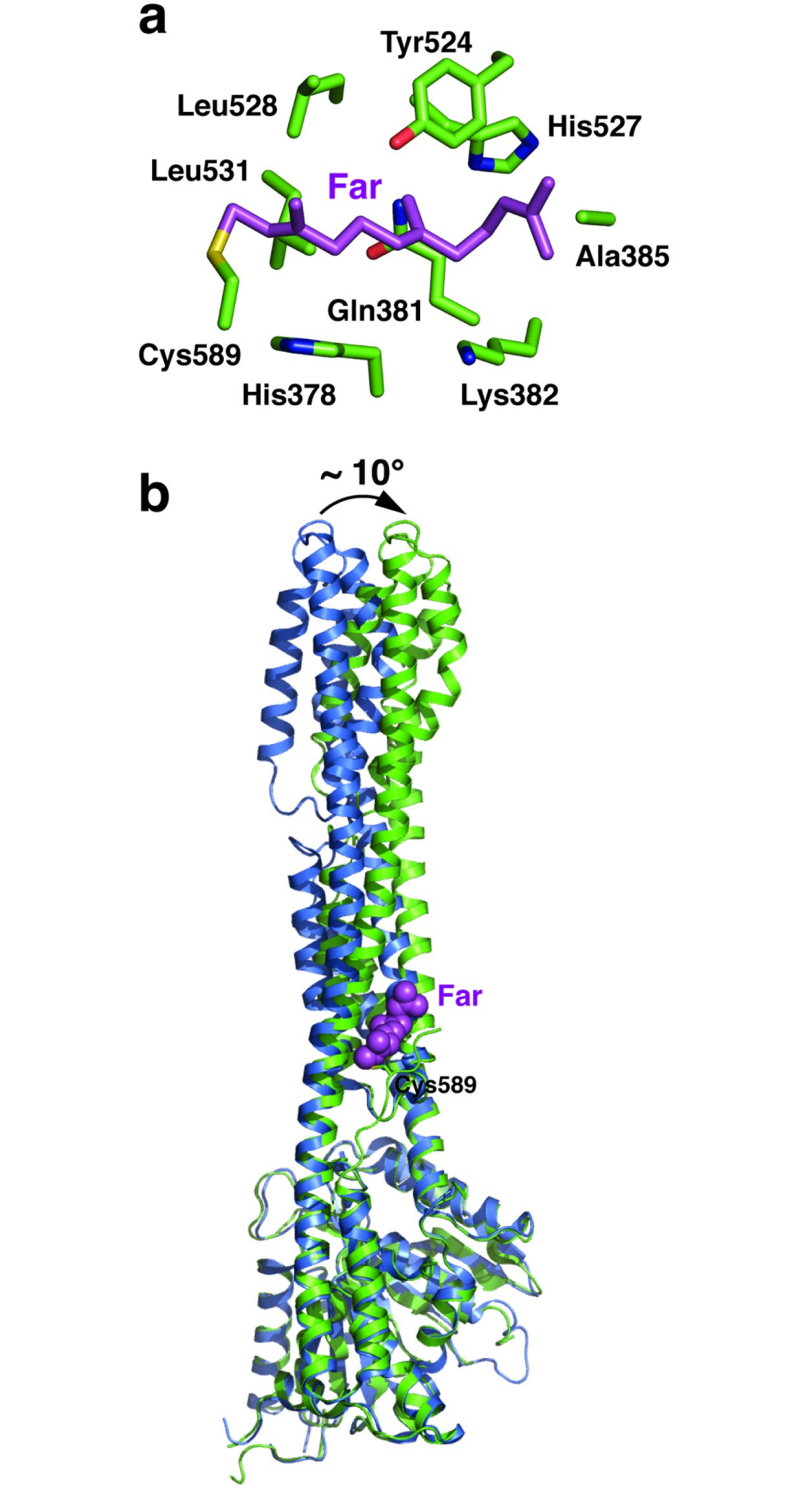
Structure of farnesylated GBP1. (**a**) An enlarged image of the farnesyl binding pocket. (**b**) GBP1_F_ (green) is superimposed onto apo GBP1 (blue, PDB ID: 1DG3). The farnesyl group (Far) is shown as magenta spheres. Rotation of the C-terminal region in GBP1_F_ is indicated by a curved arrow.

To assess how the farnesyl group affects GBP1 structure, we followed a previously described protocol [36] and prepared farnesylated GBP1 (GBP1_F_) by co-expressing GBP1 with the farnesyltransferase in *E*. *coli*. Successful modification is confirmed by mass spectrometry analyses of the purified protein (S6a Fig). We subsequently determined the crystal structure of GBP1_F_ (Table 1). Interpretable electron density is present for the farnesyl group, as well as the entire C-terminal tail of GBP1 (S6b Fig). The farnesyl group is accommodated in a pocket formed by His378, Gln381, Lys382, Ala385 from the α9 helix and Tyr524, His527, Leu528, Leu531 from the α12 helix (Fig 7a). These interactions pull the α12 helix towards the α9 helix, and cause the GED domain to become more tightly fastened to the rest of the protein. In this conformation, the α7 helix remains bent; while the N-terminal half the α12 helix, as well as the majority of the MD domain, undergoes a ~10° rotation when compared to the previously determined full-length GBP1 structure (Fig 7b).

## Discussion

Despite the fact that GBP1 was identified more than 30 years ago as one of the most prominent proteins that are induced by the interferons, its precise function remains elusive. Recent studies suggest that GBP1 inhibits intracellular bacterial replication by translocating to the bacterial surface, hindering their actin-dependent motility, and blocking their cell-to-cell spread [6–8]. Clearly, GBP1 plays an important role in cell-autonomous immunity, and poses a major threat to cytosolic bacteria such as *S*. *flexneri*. In the arms race between the bacteria and the host, *S*. *flexneri* has acquired the ability to eliminate a subgroup of GBPs through the action of its virulence E3 ligase IpaH9.8. To provide insight into the interaction between IpaH9.8 and the GBPs, we have solved the crystal structure of the LRR domain of IpaH9.8 in complex with a major fragment of GBP1. Our results show that the residues involved in interacting with GBP1 are unique to IpaH9.8, elucidating how IpaH9.8, but not other IpaH family proteins, can specifically target the GBPs. Due to the differences in the Switch II and α3 helix regions, GBP3, GBP5, and GBP7 are not efficiently degraded by IpaH9.8. Mutating relevant residues in these GBPs makes the mutant proteins more susceptible to IapH9.8-mediated degradation. By comparing our structure with other IpaH proteins in complex with their target molecules, we further reveal interaction hot spots in the LRR domain of this unique family of bacterial effectors. These results provide a deeper understanding on the pathogenesis of *S*. *flexneri*, and may facilitate the investigation of other IpaH proteins in the future.

Our results also shed light on the structural dynamics of GBP1. Previously, GBP1 without the farnesyl moiety has been crystallized in the apo state and in complex with GMP-PNP, a nonhydrolyzable analog of GTP [21, 22]. However, the two structures are largely similar and have not provided sufficient insights into the conformational change of GBP1. Through the examination of the GBP1^LG-MD^ and GBP_F_ structures determined in this study, we uncovered two new conformations of GBP1. In a way, the GBP1_F_ structure likely reflects GBP1 at its most tense state. By creating additional interactions between the GED domain and the MD domain, the farnesyl group appears to function as the second tier of bolt to lock the GED domain to the rest of the protein. A bending of the α7 helix is forced in this conformation. In contrast, the GBP1^LG-MD^ structure likely reflects GBP1 at its most relaxed state. We envision that when the structural restraints imposed by the GED domain and the farnesyl group are relieved upon GBP1 activation, the α7 helix would become straight, and this would cause the C-terminal region to rotate like seen here in the GBP1^LG-MD^ structure. How the GED domain and the farnesyl moiety are arranged in the active state, and how these conformational changes are translated to the function of GBP1, remain important questions to be addressed. In this regard, it is worth noting that, GBP5ta, a splicing variant of GBP5 that is associated with the T-cell lymphoma tissues, naturally lacks the GED domain [37]. GBP3ΔC, a splicing variant of GBP3 that does not have the α13 helix, has also been reported [38]. The functional significance of these GBP variants are unclear, but they would be more prone to adopt a relaxed conformation compared to full-length GBP5 and GBP3.

## Methods

### Cloning, expression, and purification

Primers used in this study are listed in Supplementary Table 1. IpaH9.8^LRR^ (residues 22–252) [6] and GBP1^LG-MD^ (residues 1–479) were expressed as His_6_-SUMO fusion proteins in *E*. *coli* BL21(DE3). The bacterial cultures were grown at 37 °C in the Luria-Bertani (LB) medium to an OD 600 of 0.6–0.8 before induced with 0.5 mM isopropyl β-D-1-thiogalactopyranoside (IPTG) at 18 °C for overnight. The cells were collected by centrifugation and were resuspended in a lysis buffer containing 50 mM Tris-HCl, pH 8.0, 500 mM NaCl, 10 mM imidazole, 5 mM β-mercaptoethanol, and 1 mM phenylmethylsulfonyl fluoride (PMSF). The cells were then disrupted by sonication, and the insoluble debris was removed by centrifugation. The supernatant was applied to a Ni-NTA column (GE Healthcare). The column was then washed extensively with a wash buffer containing 50 mM Tris-HCl, pH 8.0, 500 mM NaCl, 30 mM imidazole, and 5 mM β-mercaptoethanol, and eluted with an elution buffer containing 50 mM Tris-HCl, pH 8.0, 150 mM NaCl, 250 mM imidazole, and 5 mM β-mercaptoethanol. Next, the eluted proteins were digested with the ULP1 protease to cleave the N-terminal His_6_-SUMO fusion tag. The protein samples were then passed through another Ni-NTA column to remove the His_6_-SUMO fusion tag and the ULP1 protease. Untagged IpaH9.8^LRR^ and GBP1^LG-MD^ were further purified by gel filtration chromatography using a Superdex 200 column (GE Healthcare), and eluted in the final buffer containing 25 mM Tris-HCl, pH 8.0, 20 mM NaCl, and 2 mM Dithiothreitol (DTT).

To obtain the farnesylated GBP1 (GBP1_F_), full-length GBP1 was cloned into a vector that is kanamycin resistant and expresses GBP1 as a His_6_-SUMO fusion protein. The two subunits of the farnesyltransferase (FTase α and β, respectively) were cloned into the pACYCDuet-1 (Novagen) vector that is chloramphenicol resistant. His_6_-SUMO-GBP1 was then co-expressed with the FTase α/β in *E*. *coli* BL21(DE3). The bacterial cultures were supplemented with both kanamycin (50 μg/ml) and chloramphenicol (25 μg/ml), and were induced with 0.5 mM IPTG at an OD 600 of 0.8. The cells were then cultured at 20°C for 18h and were collected by centrifugation. The GBP1_F_ was then purified similarly as described above for the GBP1^LG-MD^ protein.

### Crystallization

To obtain the IpaH9.8^LRR^/GBP1^LG-MD^ complex, purified IpaH9.8^LRR^ and GBP1^LG-MD^ were incubated overnight on ice using a 1.5:1 molar ratio. The mixtures were then passed through a Superdex 200 column and eluted using the final buffer described above. The protein complex was concentrated to 18 mg/ml for crystallization. Crystals were grown at 20°C using the sitting drop vapor diffusion method. The crystallization solution contains 1.6 M sodium/potassium phosphate, pH 6.5. Crystals grew to full size in several days and were transferred to a cryo solution containing 1.6 M sodium/potassium phosphate, pH 6.5, and 38% sucrose before flash-cooled in liquid nitrogen.

GBP1_F_ was crystallized using the sitting drop vapor diffusion method at a concentration of 15 mg/ml. Crystals appeared overnight in 20 mM citric acid, 80 mM Bis-Tris propane, pH 8.8, and 16% (w/v) Polyethylene glycol 3,350. For data collection, the crystals were transferred to a solution containing 20 mM citric acid, 80 mM Bis-Tris propane, pH 8.8, 16% Polyethylene glycol 3,350, and 20% ethylene glycol before flash-cooled in liquid nitrogen.

### Data collection, structure determination, and molecular dynamics

The diffraction data were collected at Shanghai Synchrotron Radiation Facility (SSRF) beamline BL17U. The diffraction data were indexed, integrated, and scaled using HKL2000 (HKL Research). The structure was determined by the molecular replacement method using the published structure of IpaH9.8^LRR^ (PDB ID:5B0N) and GBP1 (PDB ID:1DG3) as the search models. The structure modeling was performed in Coot [39] and refined using Phenix [40]. Structural validation was performed with MolProbity [41]. Composite omit map was generated with Phenix [42].

The structure models of GBP6 and GBP7 were obtained by homology modeling using MODELLER [43] with GBP1 structure as the template. The molecular dynamics simulations were carried out using the GROMACS 5.1.2 package (http://www.gromacs.org) [44].

### Cell culture, transfection, and immunoprecipitation

HEK293T and HeLa cells, originally obtained from ATCC, were grown in a humidified incubator with 5% CO_2_ at 37 °C in Dulbecco's modified Eagle's medium (DMEM) supplemented with 10% fetal bovine serum (FBS) and 100 μg/ml penicillin/streptomycin (GIBCO). All cell lines were tested to be free of mycoplasma by the standard PCR method.

The mammalian expression plasmids have been previously described [6]. Mutations were introduced into plasmids by a PCR-based method. For the immunoprecipitation experiments, a catalytically dead mutant of IpaH9.8 (IpaH9.8-C337A) was used, since wild-type IpaH9.8 would lead to quick degradation of co-expressed GBPs. HEK293T cells were grown in 10 cm dishes to 70%-80% confluency. They were then co-transfected with 5 μg IpaH9.8-C337A and 10 μg indicated GBP plasmids using Polyethylenimine (PEI). The cells were harvested 18–24 hours later, washed with the phosphate-buffered saline (PBS) buffer, and lysed in a buffer containing 25 mM Tris-HCl, pH 8.0, 2 mM MgCl_2_, 1 mM GTP, 1 mM PMSF, and 0.5% Triton X-100. The cell lysates were cleared by centrifugation, and then incubated with the Flag M2 beads (Sigma, A2220) for 2 hours. The beads were spun down and then washed three times with the wash buffer (25 mM Tris-HCl, pH 8.0, 2 mM MgCl_2_, 1 mM GTP, and 0.2% Triton X-100). The immunoprecipitated proteins were eluted from the beads using the 3x Flag peptides (NJPeptide, NJP50002) and analyzed by SDS-PAGE and western blotting. Purified GBP1 protein interacts strongly with purified IpaH9.8 under all nucleotide conditions (apo, GMP, GDP, GppNHp, and GDP-AlFx) [6]. Also, no nucleotide is required for the formation of the IpaH9.8^LRR^/GBP1^LG-MD^ complex. However, we observed more consistent binding between GBP1 and IpaH9.8 co-expressed in cells when we included GTP in the lysis buffer. The reason for this is not entirely clear. We noticed that GBP1 tends to form puncta/aggregates when overexpressed in HEK293T cells, and we hypothesized that GTP may help to solubilize these aggregates.

For the degradation experiments, HEK293T cells were grown to 70%-80% confluency in 6-well plates, and were transfected with indicated plasmids using PEI. 18–24 h after transfection, the cells were harvested, washed, and then lysed in a lysis buffer containing 25 mM Tris-HCl, pH 8.0, and 0.5% Triton X-100. The cell lysates were cleared by centrifugation and then analyzed by western blot using antibodies for HA (Sigma, H3663), c-Myc (HuaxingBio, HX1802), Flag (Sigma, F3165), and β-tubulin (TransGen, HC101).

### Bacteria strain and cell infection

The IpaH9.8 gene with indicated mutations were cloned into the pME6032-10x SunTag plasmid as previously described [6]. *S*. *flexneri ΔipaH9*.*8 2a* strains were then transformed with these plasmids, and single colonies were picked up for each individual plasmid. The bacterial strains were cultured overnight at 37°C in the LB broth, before diluted 1:100 in fresh LB broth, and grown to an OD 600 of 0.8 in the presence of IPTG.

The HeLa cell line stably expressing RFP-GBP1 and scFv-GCN4-GFP was described previously [6]. The cells were seeded onto glass coverslips in 24-well plates and cultured for 16 h before infection. The infection (MOI, 50) was facilitated by centrifugation at 800 g for 5 min at room temperature, and cultured for another hour at 37°C in a 5% CO_2_ incubator. Cells were washed three times with PBS. Fresh DMEM containing 100 μg/ml gentamycin was then added to kill the extracellular bacteria. Two hours later, infected cells were washed three times with PBS, fixed with 4% paraformaldehyde for 30 min at room temperature, and then place in the mounting medium (ZSGB-BIO, ZLI-9556) for imaging. Cell images were recorded using the Zeiss LSM 510 Meta confocal microscope and processed with the LSM software package.

## Supporting information

S1 Fig Electron density map for the GBP1^LG-MD^/IpaH9.8^LRR^ complex. (**a**) A composite omit map (blue mesh) is contoured at 1.8 σ to depict the 2mFo-DFc electron density of the GBP1^LG-MD^/IpaH9.8^LRR^ complex crystal. There is one GBP1^LG-MD^/IpaH9.8^LRR^ complex (ribbons) in the crystal asymmetric unit, and the extra densities belong to the symmetry-related molecules. (**b**) A stereo view of the map section that covers the GBP1^LG-MD^/IpaH9.8^LRR^ interface residues. The composite omit map is contoured at 1.2 σ and depicted as grey mesh. IpaH9.8 residues are shown in cyan, and GBP1 residues are shown in orange.
(TIF)

S2 Fig IpaH9.8 would disrupt the GBP1 dimer. (**a**) The GBP1^LG-MD^/IpaH9.8^LRR^ complex is superimposed onto full-length GBP1 (PDB ID: 1DG3). The GBP1^LG-MD^/IpaH9.8^LRR^ complex is colored using the same scheme as in Fig 1. Full-length GBP1 is shown in blue. The GED domain is located on the opposite side of IpaH9.8-binding site and would not interfere with the interaction. (**b**) The GBP1^LG-MD^/IpaH9.8^LRR^ complex is superimposed onto one molecule (Mol1, green) in the GBP^LG^ dimer (PDB ID: 2B8W). IpaH9.8^LRR^ would clash with the other protomer (Mol2, brown) in the dimer and therefore prevent the dimer formation.
(TIF)

S3 Fig Structure-based sequence alignment of the IpaH proteins. Secondary structures of IpaH9.8 are shown above the sequence blocks. Residues that involved in interacting with the target proteins in IpaH9.8, SspH1, and Slrp are shaded in yellow. The three hot spot residues are shaded in red and highlighted with red asterisks.
(TIF)

S4 Fig Sequence alignment of the GBPs. The secondary structures of GBP1 are shown above the sequence blocks and labeled following the convention described in Ref. [21]. The three regions involved in binding to IpaH9.8 (P-loop, Switch II, and the α3 helix) are highlighted with orange rectangles. Residues in GBP3, GBP5, and GBP7 that likely contribute to reduced interaction with IpaH9.8 are highlighted in red and underlined. The highly conserved Gln (Gln321 in GBP1) that may be involved in regulating the conformation of the α7 helix is shaded in red.
(TIF)

S5 Fig MD simulations suggest that the α3 helix region of GBP7 prefers to adopt a loop conformation. (**a**) The structure models of GBP6 and GBP7 are obtained by homology modeling using the GBP1 structure as the template. The Switch II and α3 helix regions in the GBPs are highlighted in red. (**b**) After energy minimization and an equilibrium simulation of 200 ns (under constant temperature and pressure, with a simulation box size of 110 Å x 180 Å x160 Å that contains 90,000 explicit waters and a total of 310,000 atoms) in molecular dynamics simulations, the α3 helix region in GBP7 turns into a loop. In contrast, the α3 helix regions in GBP1 and GBP6 remain as helices. The secondary structures are annotated with the Dss algorithm in PyMol (Schrödinger, LLC). In the three energy-minimized and equilibrated structures, the average hydrogen bonding distances between residue S113 and residue E147 (over the last 100 ns MD simulation) are 6.45 ± 1.14 Å, 6.27 ± 1.36 Å, and 2.56 ± 1.03 Å in GBP1, GBP6 and GBP7, respectively. The strong interaction between S113 and E147 in GBP7 may contribute to the unfolding of α3. (**c**) In contrast to wild-type GBP7, the helical conformation of α3 in GBP7-M persists after energy minimization and equilibration MD simulation.
(TIF)

S6 Fig Farnesylation of GBP1. (**a**) Electrospray ionization mass spectrometry of unmodified and farnesylated GBP1. (**b**) The 2mFo-DFc electron density map (1.0 σ) for the C-terminal region of GBP1_F_ is shown, revealing the presence of the farnesyl group.
(TIF)
