# Supplementary figures and images for: Structural mechanism for guanylate-binding proteins (GBPs) targeting by the Shigella E3 ligase IpaH9.8
Source: PLoS Pathog. 2019 Jun 19;15(6):e1007876. doi: 10.1371/journal.ppat.1007876 (PMC6602295; doi:10.1371/journal.ppat.1007876)

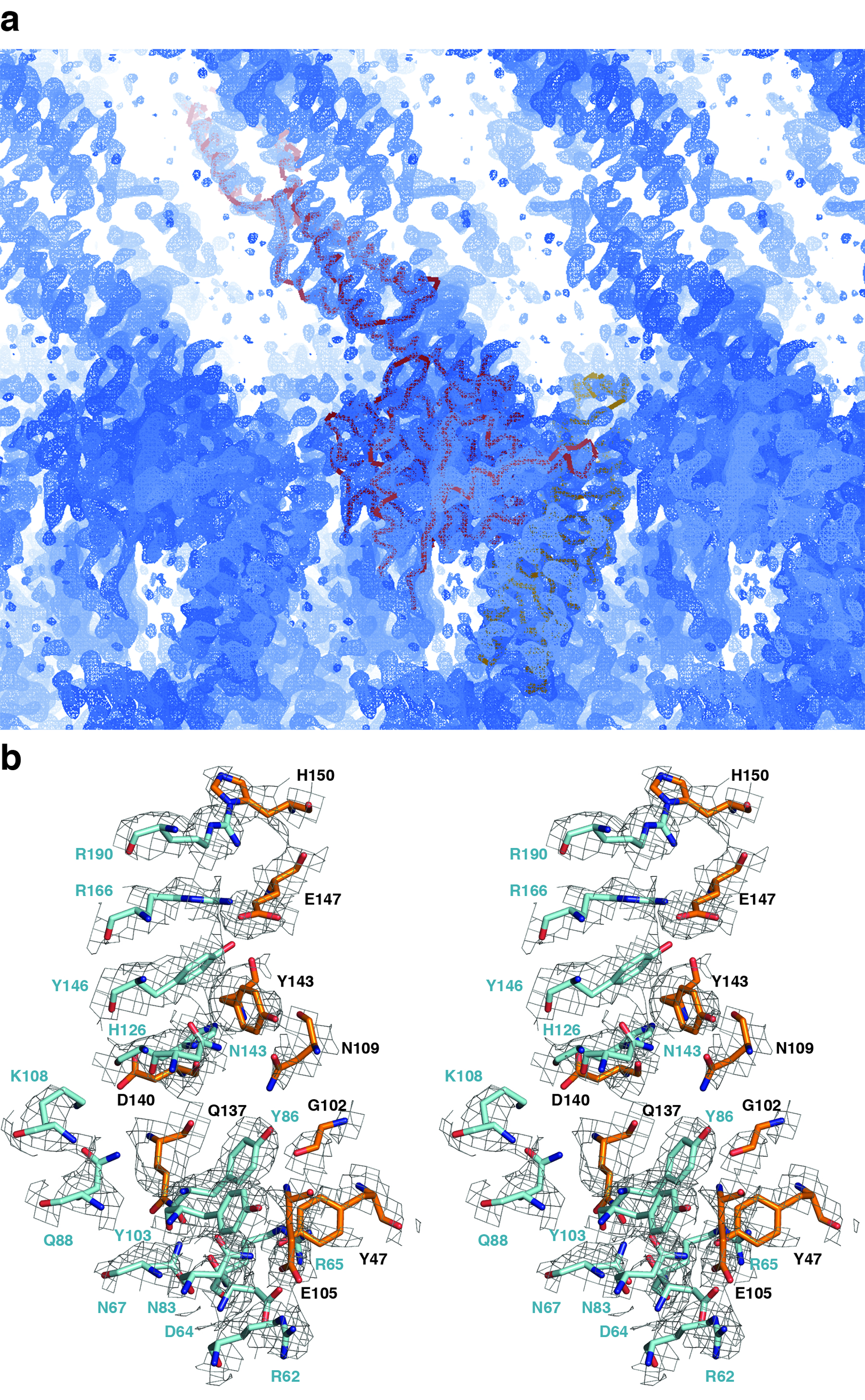

Supplement: S1 Fig — (a) A composite omit map (blue mesh) is contoured at 1.8 σ to depict the 2mFo-DFc electron density of the GBP1LG-MD/IpaH9.8LRR complex crystal. There is one GBP1LG-MD/IpaH9.8LRR complex (ribbons) in the crystal asymmetric unit, and the extra densities belong to the symmetry-related molecules. (b) A stereo view of the map section that covers the GBP1LG-MD/IpaH9.8LRR interface residues. The composite omit map is contoured at 1.2 σ and depicted as grey mesh. IpaH9.8 residues are shown in cyan, and GBP1 residues are shown in orange. (TIF) [file ppat.1007876.s001.tif]

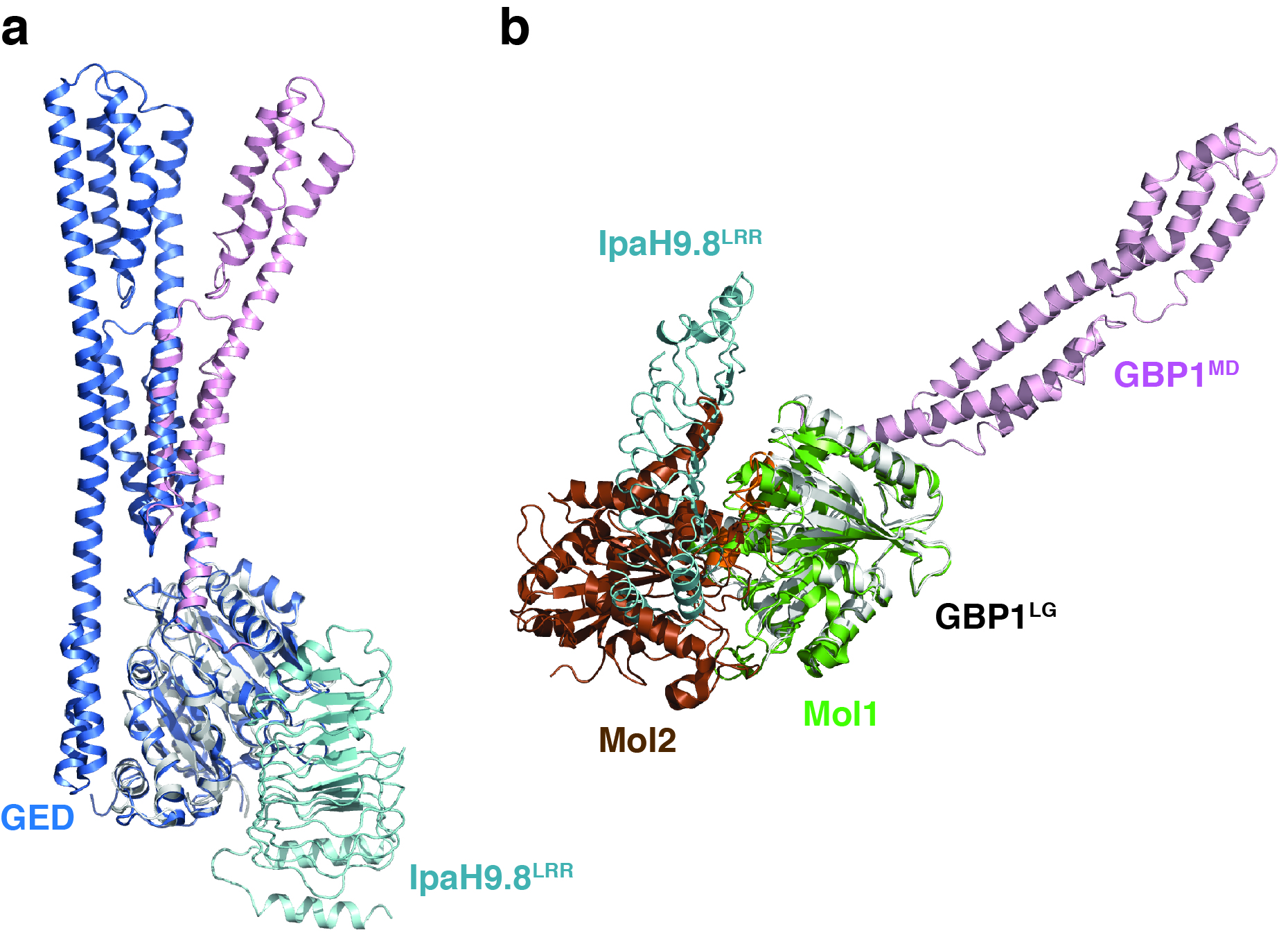

Supplement: S2 Fig — (a) The GBP1LG-MD/IpaH9.8LRR complex is superimposed onto full-length GBP1 (PDB ID: 1DG3). The GBP1LG-MD/IpaH9.8LRR complex is colored using the same scheme as in Fig 1. Full-length GBP1 is shown in blue. The GED domain is located on the opposite side of IpaH9.8-binding site and would not interfere with the interaction. (b) The GBP1LG-MD/IpaH9.8LRR complex is superimposed onto one molecule (Mol1, green) in the GBPLG dimer (PDB ID: 2B8W). IpaH9.8LRR would clash with the other protomer (Mol2, brown) in the dimer and therefore prevent the dimer formation. (TIF) [file ppat.1007876.s002.tif]

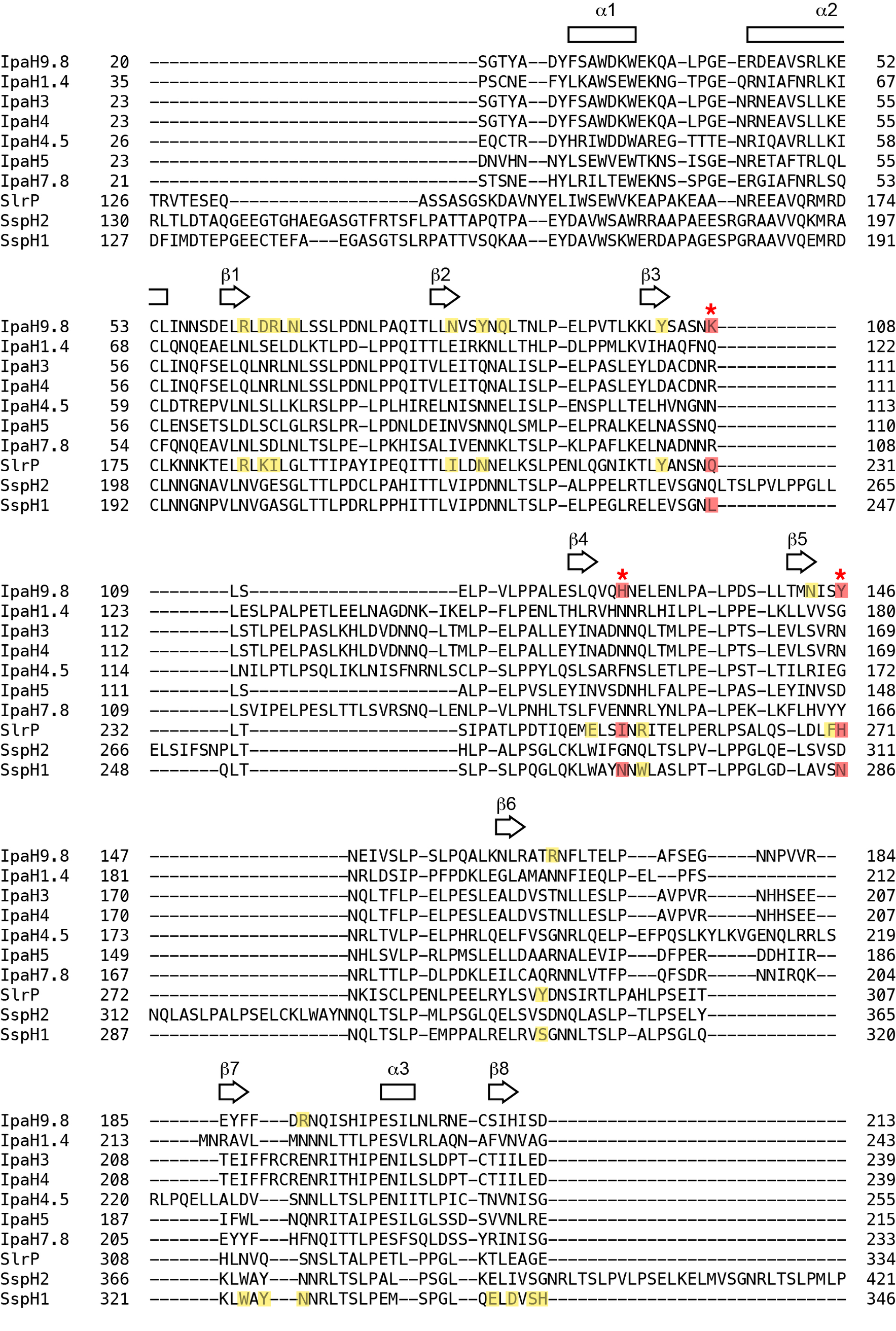

Supplement: S3 Fig — Secondary structures of IpaH9.8 are shown above the sequence blocks. Residues that involved in interacting with the target proteins in IpaH9.8, SspH1, and Slrp are shaded in yellow. The three hot spot residues are shaded in red and highlighted with red asterisks. (TIF) [file ppat.1007876.s003.tif]

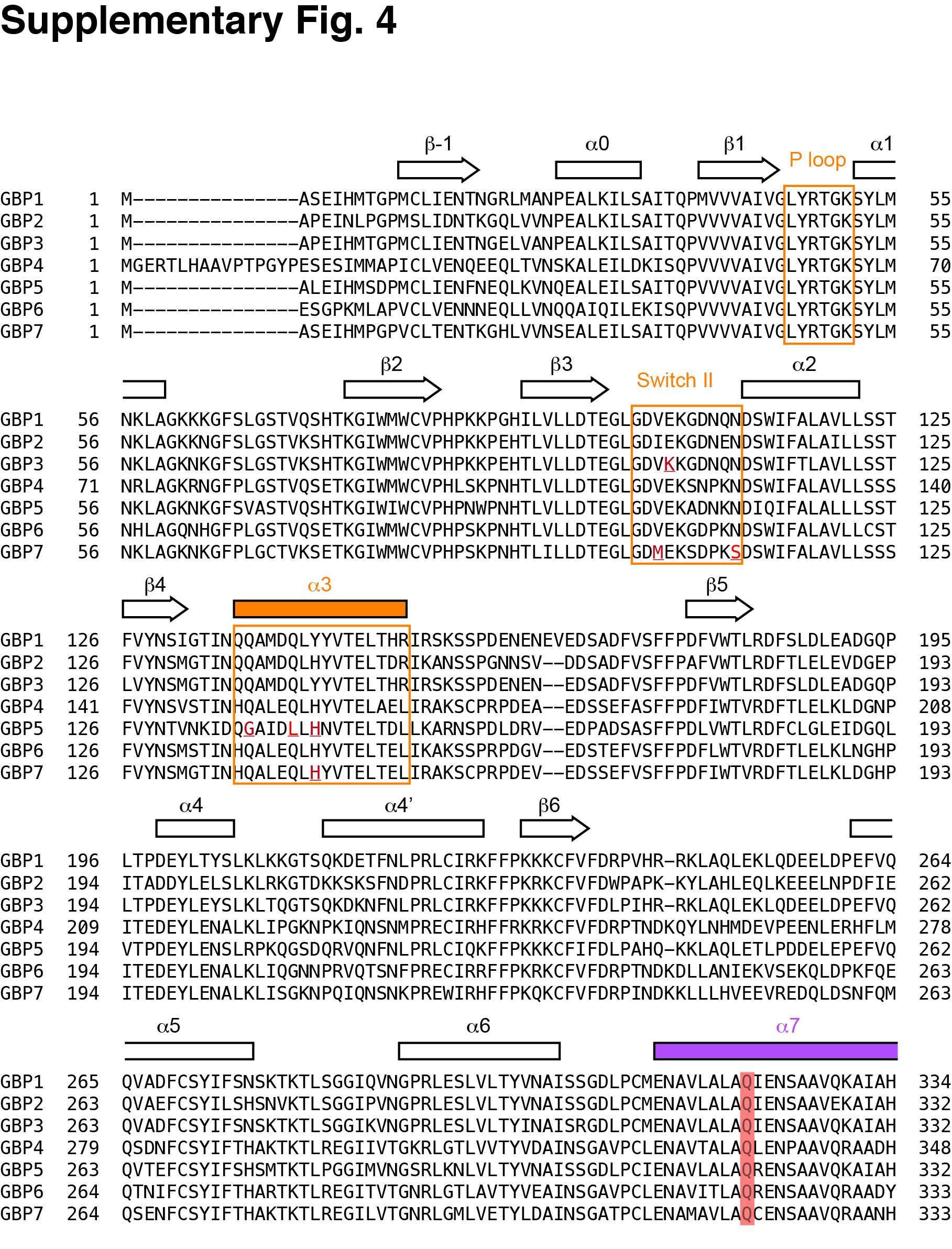

Supplement: S4 Fig — The secondary structures of GBP1 are shown above the sequence blocks and labeled following the convention described in Ref. [21]. The three regions involved in binding to IpaH9.8 (P-loop, Switch II, and the α3 helix) are highlighted with orange rectangles. Residues in GBP3, GBP5, and GBP7 that likely contribute to reduced interaction with IpaH9.8 are highlighted in red and underlined. The highly conserved Gln (Gln321 in GBP1) that may be involved in regulating the conformation of the α7 helix is shaded in red. (TIF) [file ppat.1007876.s004.tif]

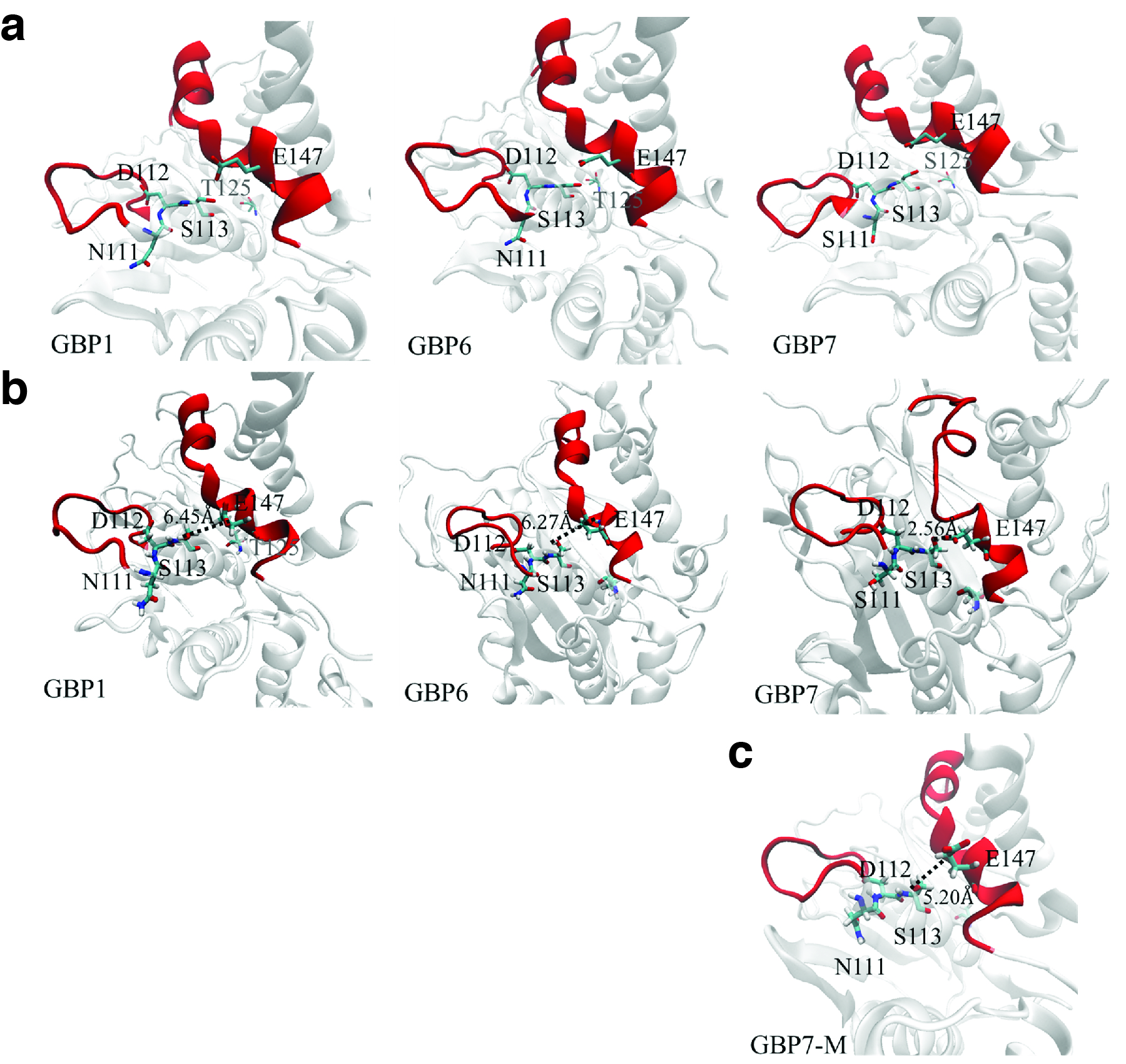

Supplement: S5 Fig — (a) The structure models of GBP6 and GBP7 are obtained by homology modeling using the GBP1 structure as the template. The Switch II and α3 helix regions in the GBPs are highlighted in red. (b) After energy minimization and an equilibrium simulation of 200 ns (under constant temperature and pressure, with a simulation box size of 110 Å x 180 Å x160 Å that contains 90,000 explicit waters and a total of 310,000 atoms) in molecular dynamics simulations, the α3 helix region in GBP7 turns into a loop. In contrast, the α3 helix regions in GBP1 and GBP6 remain as helices. The secondary structures are annotated with the Dss algorithm in PyMol (Schrödinger, LLC). In the three energy-minimized and equilibrated structures, the average hydrogen bonding distances between residue S113 and residue E147 (over the last 100 ns MD simulation) are 6.45 ± 1.14 Å, 6.27 ± 1.36 Å, and 2.56 ± 1.03 Å in GBP1, GBP6 and GBP7, respectively. The strong interaction between S113 and E147 in GBP7 may contribute to the unfolding of α3. (c) In contrast to wild-type GBP7, the helical conformation of α3 in GBP7-M persists after energy minimization and equilibration MD simulation. (TIF) [file ppat.1007876.s005.tif]

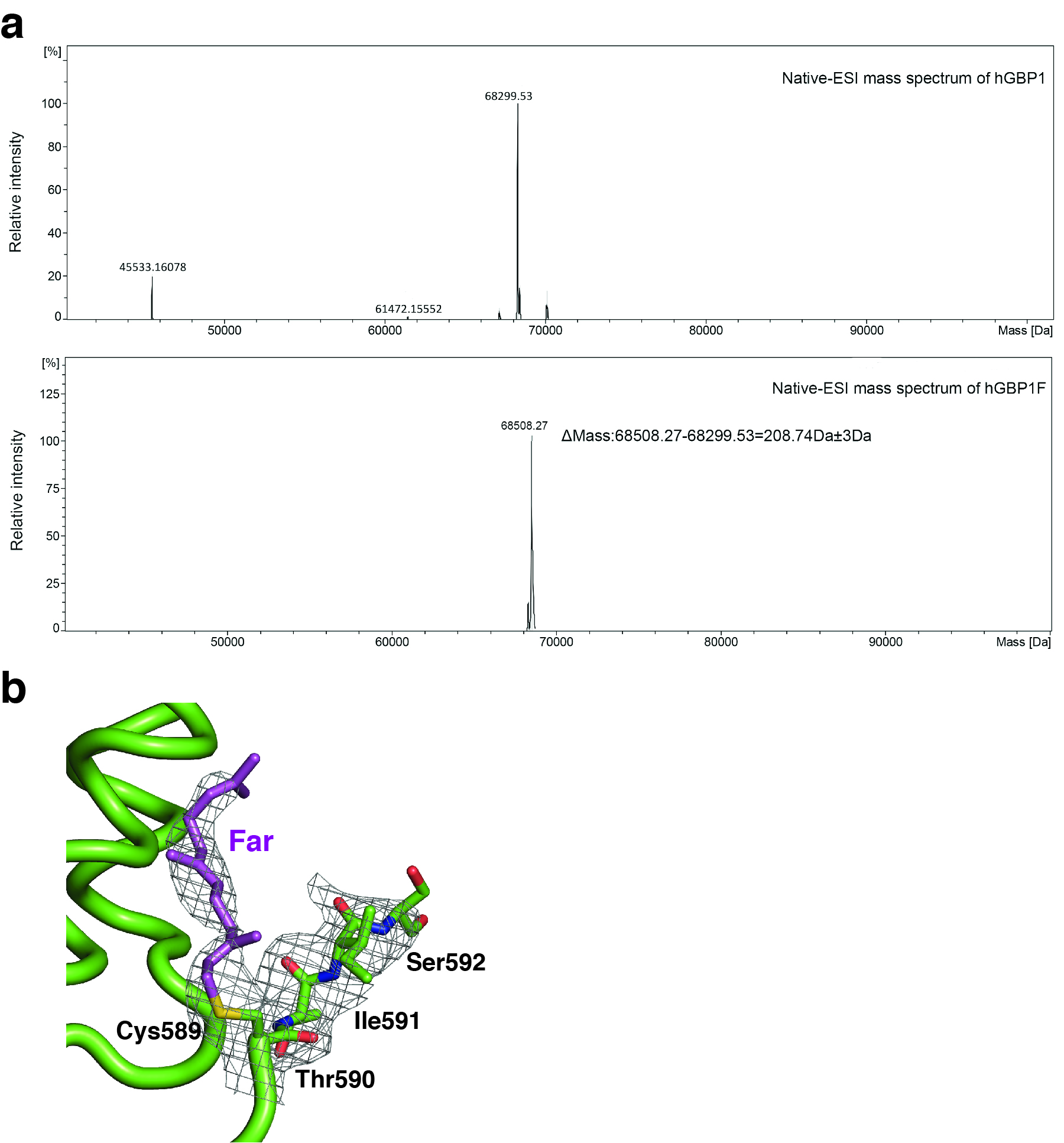

Supplement: S6 Fig — (a) Electrospray ionization mass spectrometry of unmodified and farnesylated GBP1. (b) The 2mFo-DFc electron density map (1.0 σ) for the C-terminal region of GBP1F is shown, revealing the presence of the farnesyl group. (TIF) [file ppat.1007876.s006.tif]
